# Using deep networks for knee range of motion monitoring in total knee arthroplasty rehabilitation

**DOI:** 10.3389/fbioe.2025.1691591

**Published:** 2025-11-19

**Authors:** Yi-Xiang Zhang, Qiu-Yu Wang, Tao Yang, Jia-He Wang, Hao-Tian Yin, Lei Wang, Jun Liu

**Affiliations:** 1 Department of Joints, Tianjin Hospital of Tianjin University (Tianjin Hospital), Tianjin, China; 2 Clinical College of Orthopedics, Tianjin Medical University, Tianjin, China; 3 School of Electrical and Information Engineering, Tianjin University, Tianjin, China; 4 School of Computer Science and Technology, Tiangong university, Tianjin, China; 5 Tianjin University Tianjin, Tianjin, China

**Keywords:** osteoarthritis, total knee arthroplasty, knee range of motion, deep networks, rehabilitation monitoring

## Abstract

**Background:**

Knee range of motion (ROM) is a key indicator of rehabilitation after total knee arthroplasty (TKA). Current tools, such as visual and protractor measurements, are cumbersome, imprecise, and require professional training, limiting their use in community or home settings. With the rise of smart healthcare, there is a need for a simple, accurate, and low-cost ROM assessment method that reduces healthcare burdens, enables home self-monitoring, and improves rehabilitation outcomes.

**Methods:**

A total of 1,103 knee images were collected from 1,790 patients who had undergone TKA. The images were classified into four categories: standard flexion, substandard flexion, standard extension, and substandard extension and six categories: 0°, 25°, 50°, 75°, 100°, and 125°. The images were processed using KROMNet, which was trained with a deep learning architecture that included convolutional, dilated convolution, channel attention layers, and fully connected layers. The model was trained and evaluated using a dataset split into training and test sets, and its performance was assessed with precision, recall, F1-score, and accuracy metrics for both the four-class and six-class tasks.

**Results:**

KROMNet achieved an accuracy of 95.02% in the four-class task and 94.12% in the more challenging six-class task. In the four-class task, the precision, recall, and F1-score were 95.04%, 94.96%, and 94.98%, respectively. In the six-class task, KROMNet demonstrated an accuracy of 94.12%, with precision, recall, and F1-scores of 94.64%, 94.59%, and 94.60%, respectively. The model’s performance was compared with other state-of-the-art methods, including Hazra’s, Du’s, Xia’s, Victoria’s, and Shiwei Liu’s models, with KROMNet consistently outperforming these models in both four-class and six-class tasks.

**Conclusion:**

The KROMNet model proposed in this study offers an accurate, efficient, cost-effective, and remotely deployable solution for monitoring knee ROM after TKA. KROMNet not only demonstrates superior recognition performance under small sample conditions but also shows strong clinical utility and potential for wider adoption, making it especially suitable for grassroots, community, and home rehabilitation settings. KROMNet is expected to become a key tool in the intelligent rehabilitation system, helping healthcare reduce costs, increase efficiency, and improve patient experience and rehabilitation quality.

## 1 Introduction

Osteoarthritis (OA) is a degenerative joint disease that leads to joint pain and dysfunction (Tang et al., 2025; Felson et al., 2000), with knee osteoarthritis (KOA) being the most common form. KOA significantly affects both the physical and mental health of patients (Roos et al., 2011; Davison et al., 2016; Van Dijk et al., 2010). TKA is a well-established surgical procedure that enhances the quality of life for patients with end-stage KOA (Carr et al., 2012). As the prevalence of KOA rises, the demand for TKA continues to increase (Siddiqi et al., 2022; Huang et al., 2022). Post-TKA rehabilitation is critical for facilitating early and optimal functional recovery (Artz et al., 2015; Wang et al., 2019). Prevailing rehabilitation models comprise either unsupervised home-based programs or referrals to institutional settings (e.g., hospital outpatient/rehabilitation centers) for physiotherapy (Zhao et al., 2025; Mark et al., 2025). The former often yields suboptimal outcomes due to patients’ limited comprehension of rehabilitation protocols, and inadequate progress monitoring (Pua et al., 2015; Magklara et al., 2014; Buus et al., 2021). The latter frequently compromises adherence due to access limitations, high costs, and logistical constraints (Bakaa et al., 2022; Moffet et al., 2015; Pritwani et al., 2024). Overall, these limitations underscore the necessity for simple, accurate, and cost-effective rehabilitation monitoring solutions.

Postoperative knee ROM restriction is a common complication following TKA, often resulting in dysfunction and patient dissatisfaction (Pua et al., 2017; Devers et al., 2011; Ha et al., 2016). Therefore, accurate assessment of knee ROM in postoperative patients is crucial for monitoring recovery and guiding rehabilitation (Gandhi et al., 2006). After conventional TKA, knee ROM typically ranges from 110° to 120°, significantly lower than that of a healthy knee (Kurosaka et al., 2002; Aglietti et al., 1988; Rand, 1993; Sultan et al., 2003; Ranawat et al., 1997). High-flexion prostheses commonly used in clinical practice today are designed to allow a knee ROM greater than 125°, enabling the patient to perform activities such as squatting and kneeling (Kim et al., 2016; Kim et al., 2024; Kim et al., 2009a; Kim et al., 2009b). However, traditional knee ROM assessment methods, such as visual inspection and long-arm goniometers, are often inaccurate and require specialized training (Brosseau et al., 2001; Hancock et al., 2018). This approach depends on patients regularly returning to the hospital for functional assessments and rehabilitation guidance. However, frequent offline follow-ups increase the financial burden on patients and put significant pressure on hospital outpatient clinics, especially with the aging population and the rapid increase in TKA surgeries. This rehabilitation pathway is especially inconvenient for patients in remote areas or with limited mobility, resulting in delayed rehabilitation and functional limitations.

With advancements in technology, automatic assessment of Knee ROM has primarily followed two research paths: The first focuses on angle prediction (regression/pose estimation), utilizing human keypoint detection, bone segment geometric modeling, or end-to-end regression networks to directly output continuous angles (Ge et al., 2025; Molteni and Andreoni, 2025; Su et al., 2025; Henry et al., 2024; Verhoeven et al., 2025). This approach enables error and consistency evaluation through metrics such as MAE, RMSE, and Bland-Altman plots. Its advantages include precise quantification and seamless integration with biomechanical analysis. However, it is sensitive to labeling quality, viewing angles, obstructions, and soft tissue deformation, and faces challenges in robustness and cross-domain generalization, particularly in small sample and uncontrolled home-based scenarios (Ge et al., 2025; Molteni and Andreoni, 2025). In addition to visual methods, wearable sensors such as Surface Electromyography (SEMG) and Inertial Measurement Units (IMU) enable remote monitoring but require professional setup and high patient cooperation, limiting their clinical applicability (Kumar et al., 2021; Wang et al., 2022; Ma et al., 2020; Cho et al., 2020; Feng et al., 2025; Pugliese et al., 2025; Han et al., 2025). Systems using inertial and optical markers provide high measurement accuracy but are associated with high costs and time consumption (López-Nava and Muñoz-Meléndez, 2016; van der Straaten et al., 2018; Wagner, 2018; Fong and Chan, 2010; Filippeschi et al., 2017). Markerless motion capture using multi-camera 3D reconstruction shows potential for ROM assessment but remains limited by accuracy and implementation challenges (McGinley et al., 2009; Gorton et al., 2009).

KROMNet provides a cost-effective and easy-to-implement solution for precise monitoring of total knee replacement patients, designed to enable automated classification of postoperative knee range of motion assessments. The developed framework achieves evaluation through handheld camera or phone, eliminating the need for specialized equipment or trained personnel, thereby effectively overcoming implementation barriers inherent in existing technologies. Notably, the proposed approach demonstrates robust performance even with limited training data, effectively addressing the challenge of accurate ROM monitoring under small-sample conditions. By simplifying clinical assessment workflows and enabling reliable remote self-monitoring, this approach aims to enhance postoperative rehabilitation management efficiency and ultimately improve long-term functional recovery in TKA patients.

## 2 Methods

### 2.1 Ethical approval and patients selection

**FIGURE 1:**
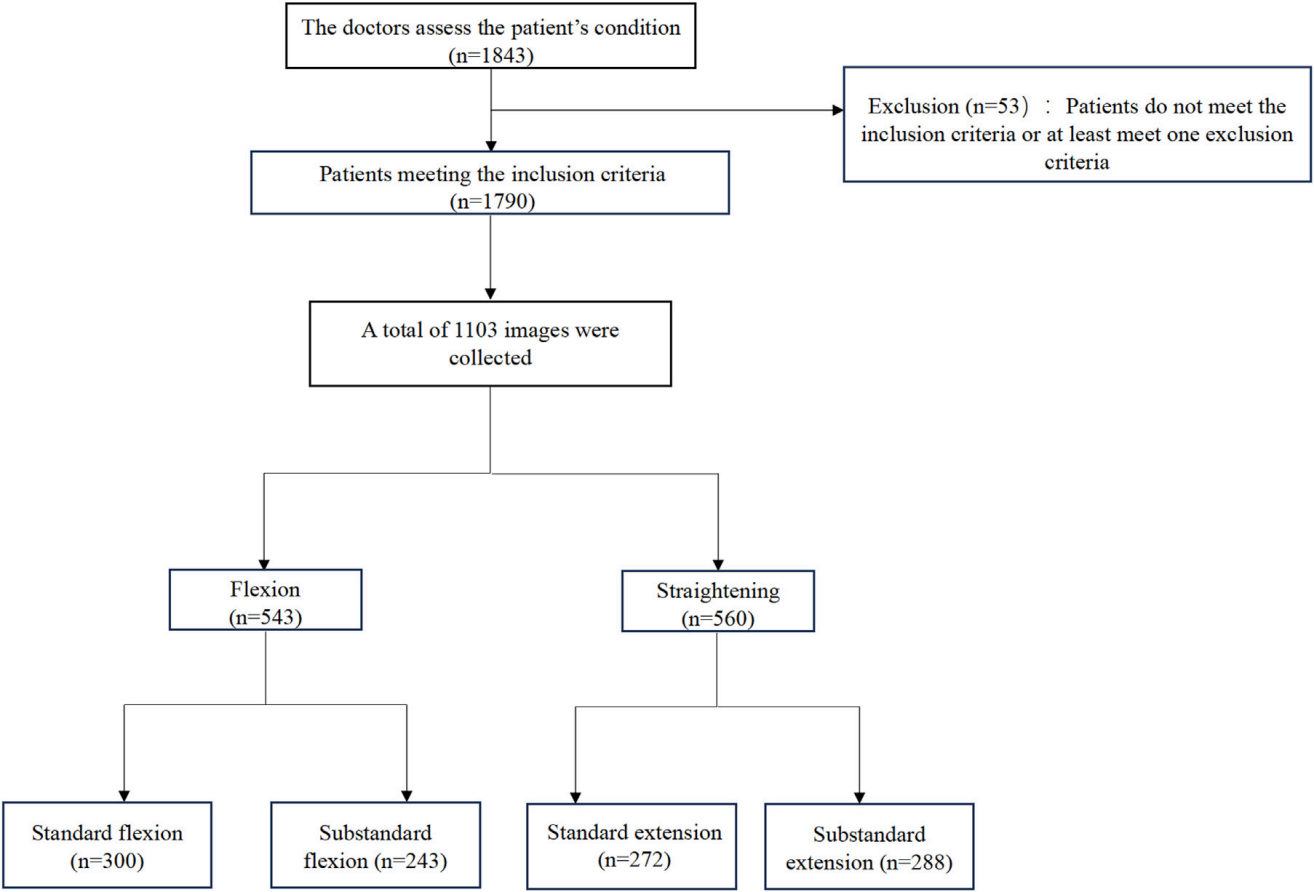
Patient enrolment and image collection process.

This study was approved by the Institutional Review Board (IRB) of Tianjin Hospital (IRB 2024 Medical Ethics Review 213) and obtained written informed consent from all participants. All image data were anonymized to ensure patient privacy. Participants were recruited from the orthopedic outpatient clinic at Tianjin Hospital between April 2024 and January 2025. A total of 1,843 patients scheduled for primary unilateral TKA were included. After applying the exclusion criteria, 1,790 patients were included. Eligible participants were aged 50–80 years and diagnosed with primary KOA by two experienced surgeons. The exclusion criteria were as follows: (1) prior lower extremity or spine injury or surgery; (2) hip, spine, or ankle diseases (including OA); (3) recent lower extremity trauma or intra-articular therapy (within 3 months); (4) frequent use of assistive devices; and (5) conditions affecting physical function, such as depression or neurological disorders (Figure 1).

### 2.2 Data collection and image acquisition

After signing the informed consent, patients underwent TKA under regional block or lumbar anesthesia and were provided with high-flexion prostheses. For patients with severe damage to the patellar articular surface, the surface was trimmed as necessary. Before discharge, patients were given rehabilitation instructions and training by a rehabilitation physician.

At the 3-month postoperative follow-up, knee was photographed at the patient’s residence under researcher supervision. The procedure was as follows: patients were instructed to lie on a clean bed, wear appropriately sized trousers, and position the operated leg outward, flexing or straightening it as much as possible without external force. Images are captured using handheld camera or phone, and the patient uploads the resulting pictures to the researcher. All uploaded images are then transferred to a cloud-based storage platform designated by the researcher for subsequent data analysis and storage. This image acquisition method in home settings effectively simulates the patient self-assessment environment, providing a data foundation for KROMNet’s future deployment on smartphones or remote rehabilitation platforms, ensuring strong real-world scalability.

**FIGURE 2:**
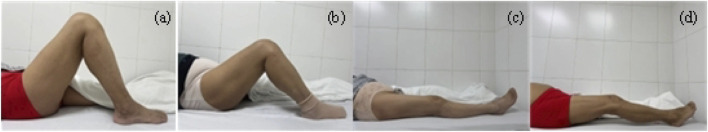
Schematic diagram of the patient’s knee ROM. **(a)** Standard flexion. **(b)** Substandard flexion. **(c)** Standard extension. **(d)** Substandard extension.

All patient-submitted photographs were thoroughly assessed for quality by the research team before being classified by radiologists. To ensure the quality and consistency of data for model training, the images were screened based on predefined criteria. The exclusion criteria were as follows: (1) Images that were excessively blurred and failed to clearly display the knee joint contour; (2) Primary knee joint regions obstructed by clothing or other objects; (3) Posture of the patient not adhering to the guidelines (e.g., legs not fully isolated, external force assistance); (4) Incorrect camera angle that was not perpendicular to the knee joint sagittal plane; (5) Insufficient lighting or overexposure, compromising image quality. Initially, we collected 2,297 images from 1,790 participants’ flexion and extension attempts. After applying the quality control criteria, 1,145 images were excluded. The remaining 1,152 high-quality images were classified by radiologists according to the predefined ROM categories. During the final dataset preparation phase for model training, an additional 49 images were excluded after being identified as duplicates or outliers during preprocessing. This resulted in a final dataset of 1,103 images for the study. The remaining photographs were classified independently by two experienced radiologists. In the event of a disagreement, a senior radiologist made the final decision. The images were initially categorized into two main groups: flexion and extension. And then further classified into four subcategories based on the Knee Society Score (KSS) (Lingard et al., 2001; Odum and Fehring, 2017): (a) Standard flexion (ROM ≥125°); (b) Substandard flexion (ROM <125°); (c) Standard extension (ROM = 0°) and (d) Substandard extension (ROM >0°) (Figure 2). A total of 1,103 photos were obtained from 1,790 participants, including 543 flexion images (300 standard and 243 substandard flexion) and 560 extension images (272 standard and 288 substandard extension) (Figure 1).

In response to the need for more detailed differentiation of patient recovery stages, we expanded the previous four-category classification into six categories. Specifically, the images were classified based on ROM thresholds of 0°, 25°, 50°, 75°, 100°, and 125°, providing a more granular representation of recovery progress. This refinement enhances the model’s ability to capture subtle variations in ROM, offering more precise clinical guidance, especially for cases where ROM falls between standard and substandard classifications. By incorporating these additional categories, we can more effectively track incremental recovery milestones, thereby improving the clinical relevance and depth of post-TKA rehabilitation assessments. The number of images in each category is as follows: 0° (139 images), 25° (206 images), 50° (219 images), 75° (221 images), 100° (204 images), and 125° (165 images). (Supplementary Figure S1).

### 2.3 Image pre-processing

The color images were first converted to grayscale, followed by binarization using Otsu’s method with automatic thresholding (Otsu, 1979; Huang et al., 2012), as shown in Equation 1. This process effectively segmented the knee region from the background, providing a reliable data foundation for subsequent classification and analysis.

$$dst\left(x,y\right)=\left\{\begin{array}{cc} \max val & \text{if }src\left(x,y\right)>thresh\\ 0 & \text{otherwise} \end{array}\right.$$ (1)

In Equation 1, src is input array; dst is output array; thresh is adaptive threshold value; max⁡val is maximum value.

### 2.4 KROMNet architecture for knee ROM assessment

**FIGURE 3:**
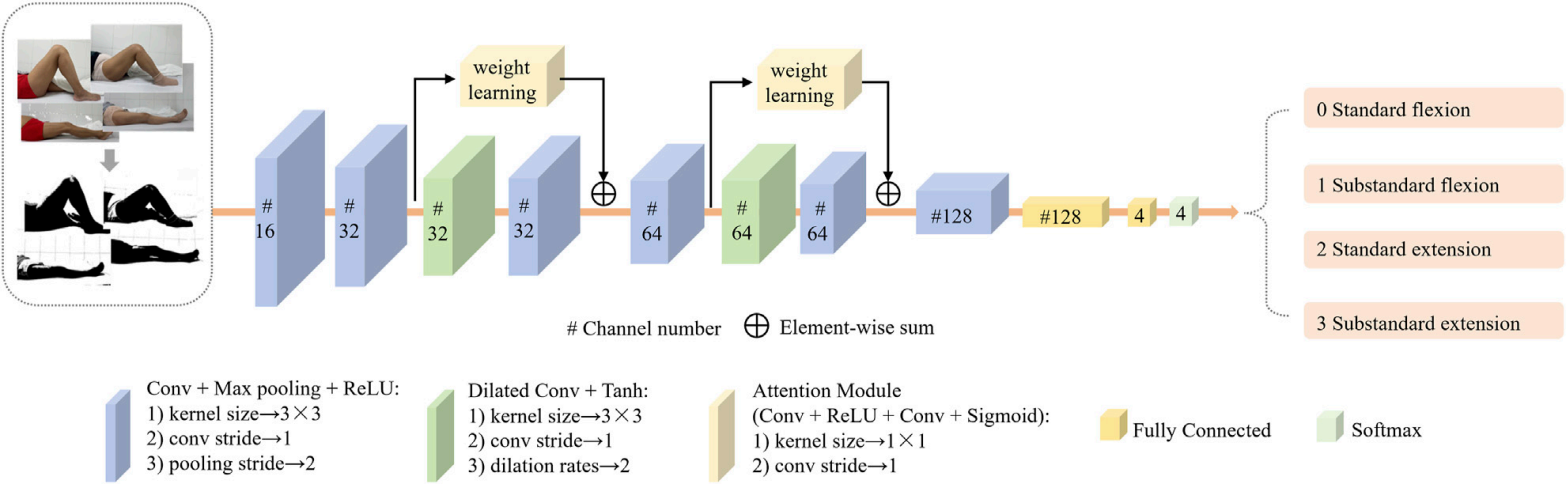
The architecture of KROMNet deep network.

The KROMNet architecture consists of six convolutional layers, two dilated convolution layers, two channel attention layers, and two fully connected layers. The input of the network are the preprocessed knee joint images. The proposed KROMNet in this article used convolutional and max-pooling layers for basic feature extraction, incorporating a dilated convolutional layer to expand the receptive field and capture multi-scale morphological features. A channel attention mechanism was integrated to enhance discriminative anatomical features adaptively. Two cascaded fully connected layers at the network’s end establish non-linear decision mapping, ultimately generating graded probability distributions of knee ROM through Softmax activation. The architectural configuration is shown in Figure 3. This architecture is designed to balance the accuracy of medical interpretation with the lightweight deployment of the model, enabling its operation in resource-limited settings and promoting the shift in knee rehabilitation assessment from “specialty-dependent” to “universal self-help.”

#### 2.4.1 Channel attention mechanism

**FIGURE 4:**
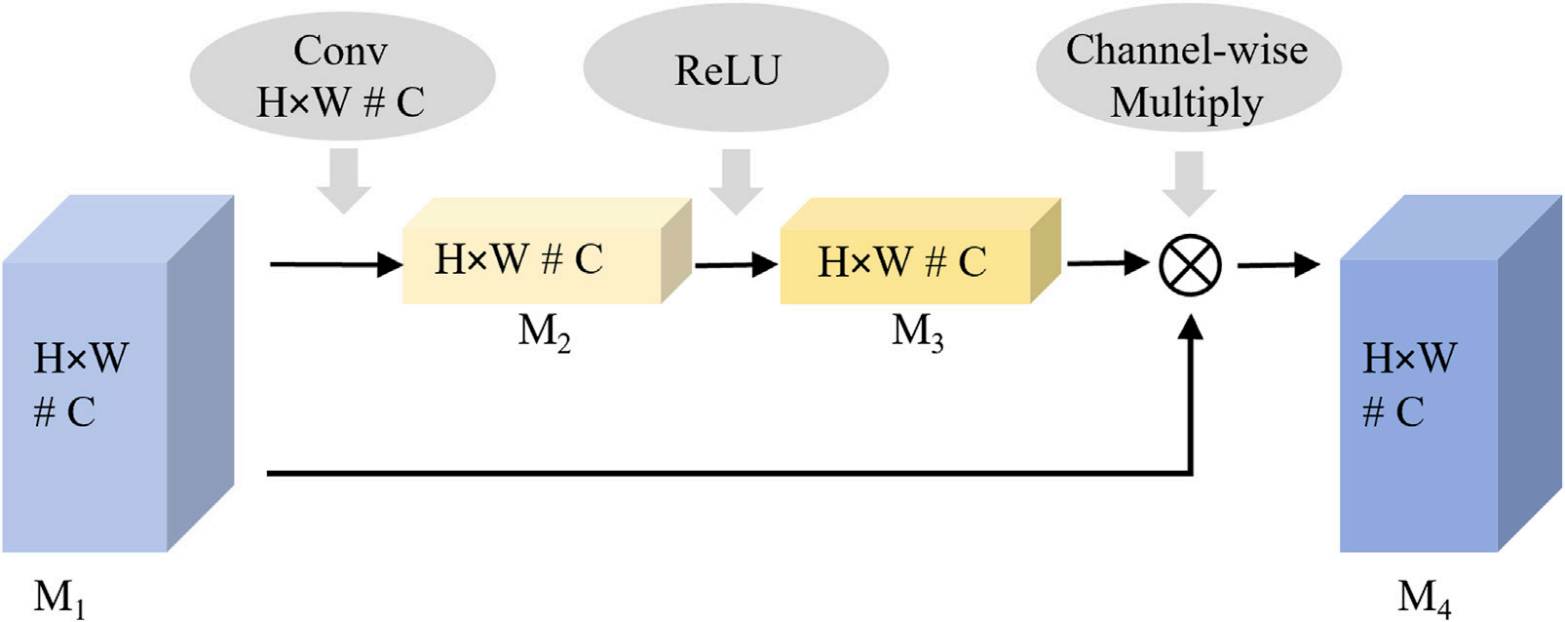
The process of the channel attention mechanism.

As shown in Figure 2, the curvature of the knee is a key distinguishing feature for evaluating knee rehabilitation. The channel attention mechanism introduced in this article enhances the network’s discriminative ability by dynamically evaluating the contribution of each channel feature in key feature learning. It assigns higher weights to more relevant channels, thereby enhancing their impact on classification while suppressing less important ones. By modeling the interdependencies between channels and recalibrating the features, the proposed network emphasizes the most discriminative data. The process of the channel attention mechanism is shown in Figure 4.

Feature significance modeling is performed by applying spatial dimensionality reduction to the input feature map *M*_1_. The two-dimensional features (*H × W*) of each channel are aggregated into one-dimensional vectors using global average pooling to extract channel-level information. This spatial aggregation process is mathematically represented in Equation 2.

$$M_{2}=\frac{1}{H\times W}\sum_{i=1}^{H}\sum_{j=1}^{W}M_{1}\left(i,j\right)$$ (2)

Weight allocation learning is performed through the fully connected layer and a nonlinear activation function, where the nonlinear relationship between channels is learned and the weight vector is generated. This weight generation mechanism is mathematically represented by Equation 3. The output value of *M*_3_ is considered the importance weight of the corresponding channel.

$$M_{3}=\sigma \left(W_{2}\cdot \delta \left(W_{1}\cdot M_{2}\right)\right)$$ (3)

Where $W_{1}\in R^{1\times 1\times C/16}$ is the descending fully-connected layer and δ is the ReLU activation function, $W_{2}\in R^{1\times 1\times C}$ is the ascending fully-connected layer and σ is the Sigmoid function.

Feature recalibration is performed by multiplying the weight feature $M_{3}$ with the original feature map $M_{1}$ channel by channel, as formulated in Equation 4, resulting in the feature map $M_{4}$ and completing the channel recalibration.

$$M_{4}=M_{1}\cdot M_{3}$$ (4)

This converts the raw $M_{1}$ into a weighted feature map, where channels with higher weights receive more attention.

#### 2.4.2 Dilated convolution

As shown in Figure 2, the local features of standard and abnormal samples are highly similar, limiting the classification performance when relying solely on local features. Therefore, integrating global contextual features of holistic lower-limb kinematic patterns while preserving fine-grained anatomical details is essential to enhance discriminative capability. Expanding the network’s receptive field becomes a key technique. Although traditional convolutional neural networks (CNN) reduce feature redundancy and enlarge receptive fields through pooling operations, two inherent limitations persist:

- Spatial resolution degradation. Pooling operations blur the spatial positional information in the feature map, hindering the precise modeling of geometric interdependencies among key anatomical structures of the leg.
- Loss of small-target information. Using a typical three-layer 2 × 2 pooling architecture as an example, after three consecutive downsamplings, structural features smaller than 8 × 8 pixels in the original image will be completely lost.

To address this challenge, the study employs dilated convolution operations that strategically insert zeros within the convolutional kernels — a mechanism distinct from subsampling approaches, expanding receptive fields while preserving spatial resolution (Yu and Koltun, 2016). The size of the receptive field is proportional to a parameter called the dilation rate; as the dilation rate increases, the number of zero-paddings also increases. When the dilation rate is set to 1, dilated convolution becomes equivalent to the traditional convolution operation. Dilated convolution retains useful information from the input without increasing network parameters and helps capture more globally representative information about the original data.

#### 2.4.3 Objective function

In classification problems, the cross-entropy loss quantifies the divergence between the ground-truth probability distribution (from expert or training data) and the model-generated probability distribution, serving as the optimization objective. The mathematical formulation of this loss function is given in Equation 5.

$$J=-\sum_{i=1}^{N}\sum_{c=1}^{K}y_{ic}\log\left({h_{\theta }\left(x_{i}\right)}_{c}\right)$$ (5)

Where N denotes the number of samples; K represents the number of categories; $y_{ic}$ indicates the one-hot encoded true label of the sample. If the true class of sample $x_{i}$ equals c , $y_{ic}$ takes 1, otherwise, it takes 0; $h_{\theta }{\left(x_{i}\right)}_{c}$ denotes the predicted probability that observed sample $x_{i}$ belongs to class c .

## 3 Results

### 3.1 Image pre-processing results

**FIGURE 5:**
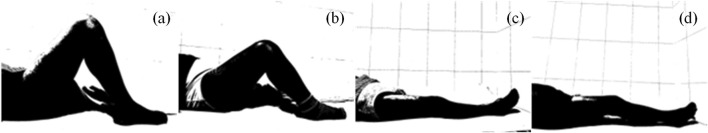
Preprocessing results of patient flexion and extension images. **(a)** Standard flexion. **(b)** Substandard flexion. **(c)** Standard extension. **(d)** Substandard extension.

The image preprocessing results, shown for the four-category classification example in Figure 5, highlight the enhancement of knee joint structures through the grayscale binarization process. The six-category classification results are presented in Supplementary Figure S2.

### 3.2 ROM assessment results based on KROMNet

The dataset labels were 0 for standard flexion, 1 for substandard flexion, 2 for standard extension, and 3 for substandard extension. The dataset consisted of 1,103 patient-derived knee joint images, which were divided into a training set and a test set by the 8:2 criterion.

**FIGURE 6:**
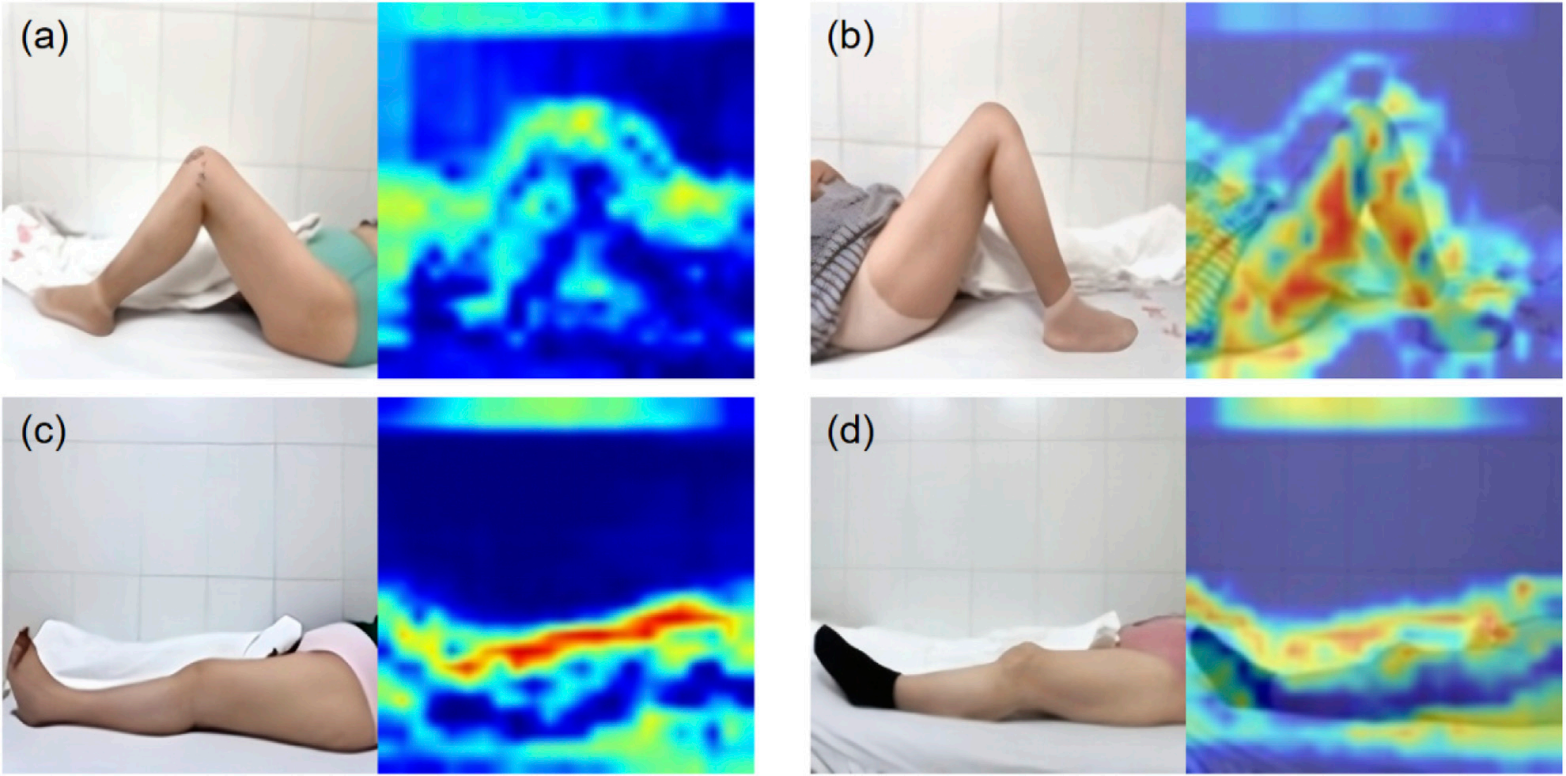
The attention maps. **(a)** Standard flexion. **(b)** Substandard flexion. **(c)** Standard extension. **(d)** Substandard extension.

The KROMNet is configured with a batch size of 16, a learning rate of 0.0002, and 100 training epochs. The binarized grayscale images are fed into the model. Figure 6 presents the attention maps derived from the channel attention mechanism in the four-category classification example, emphasizing the model’s ability to capture key anatomical features within the knee joint region.

The model’s performance was assessed using precision, recall, F1-score, and accuracy. These metrics were derived from the counts of true positives (TP), false positives (FP), true negatives (TN), and false negatives (FN) across all categories. Recall measures the model’s ability to correctly identify positive instances, while precision reflects the accuracy of its positive predictions. The F1-score, which is the harmonic mean of precision and recall, provides a balanced evaluation, particularly important when dealing with potential class imbalance. The formulas for these metrics are presented in Equations 6–9.

$$Precision=\frac{TP}{TP+FP}$$ (6)

$$Recall=\frac{TP}{TP+FN}$$ (7)

$$F1-score=2\times \frac{precision\times Recall}{precision+Recall}$$ (8)

$$Accuracy=\frac{TP}{TP+FP+FN+TN}\times 100\%$$ (9)

The proposed KROMNet model demonstrates excellent classification performance in both four-class and six-class tasks, as shown in Tables 1, 2. In the four-class task, the precision, recall, and F1-score on the training set reach 0.9943, 0.9951, and 0.9947, respectively, with an accuracy of 99.53%; on the testing set, the corresponding metrics are 0.9504, 0.9496, and 0.9498, with an accuracy of 95.02%. In the more challenging six-class task, the model also achieves outstanding results, with precision, recall, and F1-score on the training set of 0.9860, 0.9857, and 0.9858, respectively, and an accuracy of 98.44%. On the testing set, these metrics further improve to 0.9464, 0.9459, and 0.9460, with an accuracy of 94.12%. In summary, even under limited sample conditions, the KROMNet model maintains high recognition performance across classification tasks with varying numbers of categories.

**TABLE 1:** KROMNet four-category classification results.

Index	Precision	Recall	F1-score	Accuracy
Training set	0.9943	0.9951	0.9947	99.53%
Testing set	0.9504	0.9496	0.9498	95.02%

**TABLE 2:** KROMNet six-category classification results.

Index	Precision	Recall	F1-score	Accuracy
Training set	0.9860	0.9857	0.9858	98.44%
Testing set	0.9464	0.9459	0.9460	94.12%

### 3.3 Comparison and analysis of different methods

This study focuses on knee image classification, addressing feature extraction and robust recognition under small sample conditions. To validate the proposed method, four advanced image classification models are compared.

- Hazra’s model. Using 2D CNN-LSTM networks with self-attention mechanisms to enhance feature extraction (Hazra and Santra, 2019).
- Du’s model. Utilizing a channel-space attention module to focus on key regions and generates diverse samples to reduce overfitting based on physical mechanisms (Tu et al., 2017).
- Xia’s model. Improving feature extraction through spatio-temporal continuity modeling using scattering center detection and tracking algorithms (Xia et al., 2021).
- Victoria’s model. A separable CNN with depthwise and pointwise convolutions, combined with a dropout layer, reducing parameters and prevents overfitting in small sample settings (Victoria et al., 2023).
- Shiwei Liu’s model. ConvNeXt leverages modern CNN architecture with design modifications inspired by Vision Transformers (ViTs) to enhance image classification performance. It improves efficiency by replacing traditional CNN blocks with layers designed to better capture fine-grained features while still maintaining the computational efficiency of CNNs (Liu et al., 2024).

### 3.4 Performance analysis of the four-class classification task

Table 3 provides a comparative analysis of training and test set accuracy among different methods for the four-class classification task. As shown in Table 3, for the four-class classification task, the KROMNet model achieves the highest test accuracy (95.02%), which is significantly higher than that of existing methods, including Hazra (92.76%), Du (91.40%), Xia (90.50%), Victoria (89.14%), and Shiwei Liu (92.76%). It is noteworthy that KROMNet also achieves a training accuracy of 99.53%, which indicates its strong learning ability and the absence of obvious overfitting.

**TABLE 3:** Training and testing accuracy of four-class classification for different methods.

Accuracy	Hazra	Du	Xia	Victoria	Shiwei Liu	KROMNet
Training set	99.19%	99.65%	86.51%	92.21%	95.47%	99.53%
Testing set	92.76%	91.40%	90.50%	89.14%	92.76%	95.02%

**FIGURE 7:**
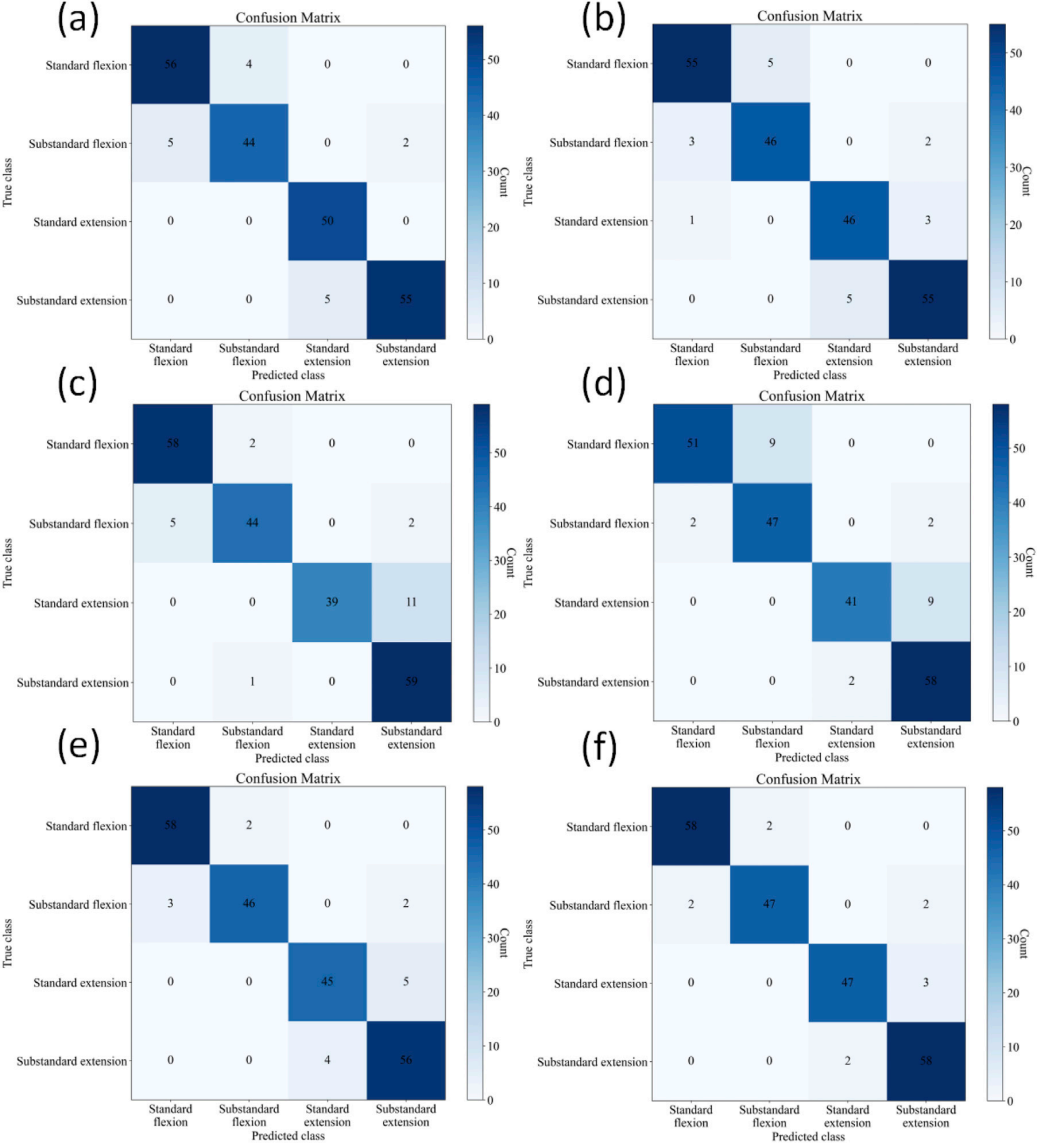
Confusion matrices of different methods on the four-class test set. **(a)** Hazra's model. **(b)** Du's model. **(c)** Xia's model. **(d)** Victoria's model. **(e)** Shiwei Liu's model. **(f)** KROMNet model.

The confusion matrices for the four-class classification using different methods are shown in Figure 7. Table 4 further presents in detail the classification performance metrics of different methods on the four-class test set. KROMNet performs best across all evaluation metrics, achieving a precision of 0.9504, a recall of 0.9496, an F1-score of 0.9498, and an accuracy of 95.02%. Compared to the suboptimal Shiwei Liu’s method, which achieves an accuracy of 92.76% with all other metrics below 0.93, KROMNet exhibits a comprehensive and significant performance improvement.

**TABLE 4:** Classification results of four-class classification on the test set for different methods.

Index	Hazra	Du	Xia	Victoria	Shiwei Liu	KROMNet
Precision	0.9272	0.9132	0.9297	0.8990	0.9291	0.9504
Recall	0.9282	0.9139	0.8982	0.8896	0.9255	0.9496
F1-score	0.9268	0.9135	0.9076	0.8903	0.9270	0.9498
Accuracy	92.76%	91.40%	90.50%	89.14%	92.76%	95.02%

### 3.5 Performance analysis of the six-class classification task

Table 5 provides a comparison of accuracy among different methods for the six-class classification task. In this more challenging six-class classification task, KROMNet achieves a test accuracy of 94.12% - a value comparable to that of the top-performing methods (Shiwei Liu, 92.55%) - and significantly outperforms Hazra and Xia (both 89.80%), Du (89.02%), and Victoria (87.84%).

**TABLE 5:** Training and testing accuracy of six-class classification for different methods.

Accuracy	Hazra	Du	Xia	Victoria	Shiwei Liu	KROMNet
Training set	94.33%	96.00%	98.67%	95.88%	99.22%	98.44%
Testing set	89.80%	89.02%	89.80%	87.84%	92.55%	94.12%

**FIGURE 8:**
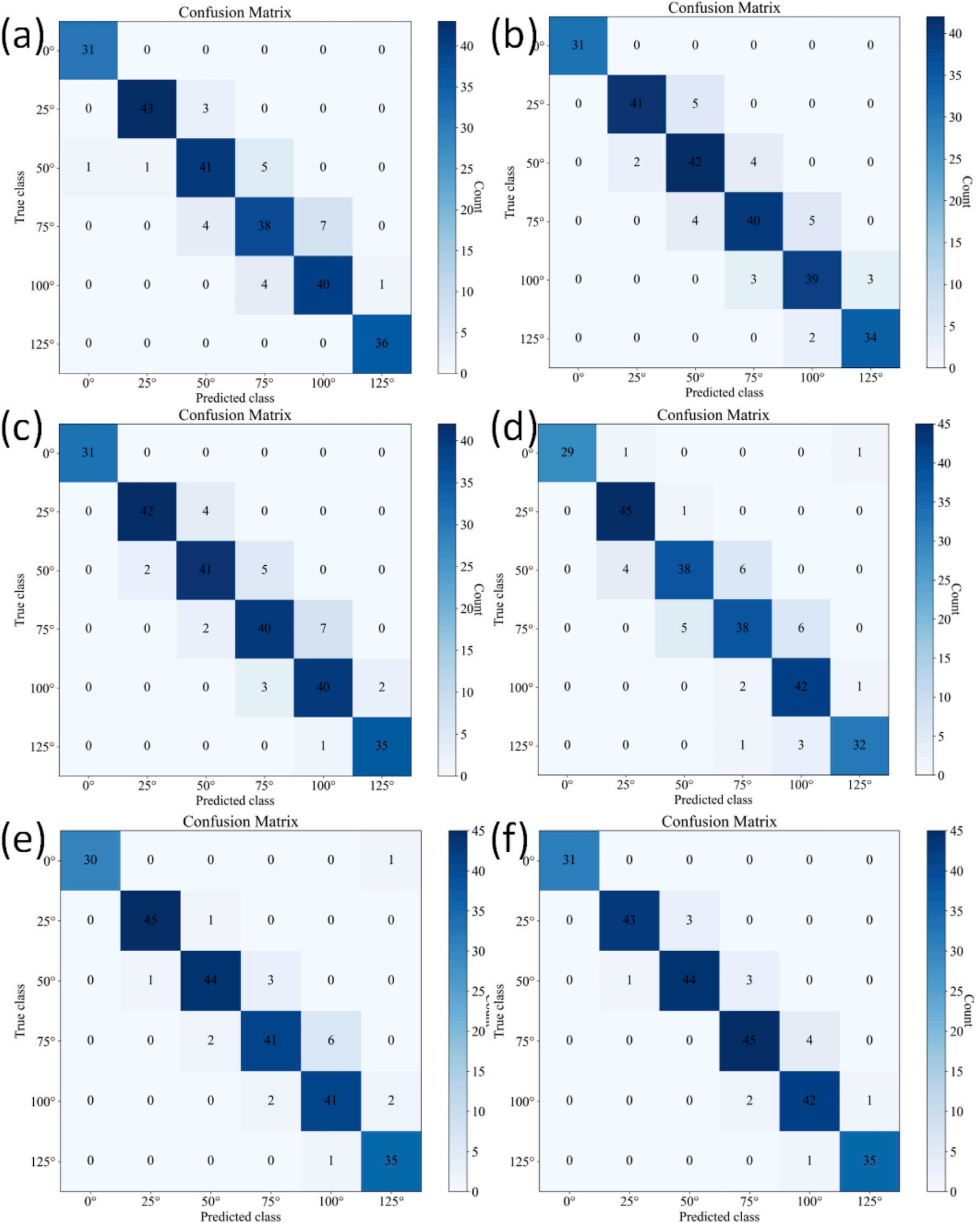
Confusion matrices of different methods on the six-class test set. **(a)** Hazra's model. **(b)** Du's model. **(c)** Xia's model. **(d)** Victoria's model. **(e)** Shiwei Liu's model. **(f)** KROMNet model.

The confusion matrices for the six-class classification test sets using different methods are shown in Figure 8. Table 6 further provides a detailed performance comparison of different methods on the six-class test set. KROMNet delivers competitive performance across a precision of 0.9464, a recall of 0.9459, an F1-score of 0.9460, and an accuracy of 94.12%. This indicates that KROMNet has achieved the state-of-the-art performance level in this complex six-class classification task.

**TABLE 6:** Classification results of six-class classification on the test set for different methods.

Index	Hazra	Du	Xia	Victoria	Shiwei Liu	KROMNet
Precision	0.9055	0.8991	0.9066	0.8895	0.9302	0.9464
Recall	0.9089	0.8990	0.9074	0.8839	0.9305	0.9459
F1-score	0.9069	0.8986	0.9067	0.8852	0.9298	0.9460
Accuracy	89.80%	89.02%	89.80%	87.84%	92.55%	94.12%

## 4 Discussion

This study aimed to develop and evaluate the KROMNet model for assessing knee ROM in patients who have undergone TKA. The results indicate that the proposed method achieved high accuracy in monitoring of knee ROM, overcoming several limitations of traditional knee ROM assessment techniques.

Our findings show that the KROMNet model achieved an accuracy of 95.02% in the four-class task and 97.28% in the six-class task, despite the small sample size. This performance is further supported by the confusion matrix and key evaluation metrics, including precision, recall, and F1-score. The model’s ability to accurately distinguish between categories in both the four-class and six-class tasks demonstrates its effectiveness in assessing knee ROM during postoperative rehabilitation. These results highlight KROMNet’s robustness across both simpler and more complex classification tasks, making it a reliable tool for monitoring knee recovery. These findings are consistent with previous research, which emphasizes the critical role of accurate knee ROM measurement in improving functional recovery and patient satisfaction after TKA (Gandhi et al., 2006). Compared to traditional methods, such as visual inspection and long-arm goniometers, the KROMNet model provides distinct advantages. Conventional techniques rely on clinician expertise, are prone to inter-observer variability, and are time-consuming, often resulting in inconsistent and inaccurate results (Brosseau et al., 2001; Hancock et al., 2018). In contrast, KROMNet provides a scalable, automated solution that eliminates the need for specialized equipment and reduces the healthcare burden associated with manual assessments.

### 4.1 Clinical justification for ROM thresholds

A key aspect of our approach is adopting the KSS thresholds for classifying knee ROM, specifically ≥125° for flexion and 0° for extension. The selection of the 125° flexion threshold is based on clear clinical rationale. After conventional TKA, knee ROM typically plateaus between 110° and 120°, which is often insufficient for demanding daily activities (Kurosaka et al., 2002; Aglietti et al., 1988; Rand, 1993; Sultan et al., 2003; Ranawat et al., 1997). In contrast, the high-flexion prostheses used in this cohort are specifically designed to achieve a ROM greater than 125° (Kim et al., 2016; Kim et al., 2024; Kim et al., 2009a; Kim et al., 2009b). The target of >125° is not arbitrary; it is functionally critical, enabling patients to perform essential high-flexion activities, such as squatting and kneeling, which are crucial for satisfaction and quality of life, especially in certain cultural and occupational contexts (Devers et al., 2011; Ha et al., 2016). Similarly, achieving full extension (0°) is biomechanically crucial, as even a slight flexion contracture can lead to an abnormal gait, increased energy expenditure, and joint instability (Gandhi et al., 2006). Although rehabilitation is a continuous process, the binary classification based on these well-established thresholds offers a clinically meaningful distinction between patients who have regained functionally adequate ROM and those who may need further intervention. This makes our assessment tool highly relevant to functional recovery goals.

### 4.2 Mechanism analysis of performance differences

The comparative analysis based on the performance metrics reveals that differences in classification effectiveness among various methods primarily stem from their core architectural designs. Conventional CNN models, due to their relatively simple structure and reliance on local convolutional kernels for feature extraction, have inherent limitations in processing complex multi-scale features and long-range dependencies, thus limiting their performance potential. While methods such as Hazra and Du enhance feature representation via attention mechanisms and multi-channel fusion, their attention-guided mechanisms and data augmentation strategies fail to strike an optimal balance between noise suppression and preservation of essential information. The Xia method, due to its multi-dimensional feature redundancy, tends to be overly sensitive to subtle variations in input data; in contrast, the Victoria method, which uses depthwise separable convolutions, sacrifices high-frequency details that are critical for accurate classification—despite reducing computational costs. In contrast, the proposed method in this work incorporates a more adaptive feature selection mechanism and a hierarchical feature fusion strategy—both of which not only strengthen the discriminative power of feature representations but also effectively suppress redundancy and noisy interference. Consequently, it consistently achieves superior and balanced performance across all evaluation metrics.

The ability to remotely and accurately monitor knee ROM is crucial, especially in post-TKA rehabilitation. The proposed KROMNet model not only achieves high-precision knee mobility assessment, but also offers a low-threshold, remote, and equipment-free solution. It overcomes the limitations of traditional rehabilitation assessments that rely on manual and professional tools, making it especially suitable for resource-limited or remote areas with high accessibility and social value.

KROMNet allows doctors to access patients’ dynamic rehabilitation data without increasing their workload, while patients can take and upload photos regularly, enabling a new model of “intelligent monitoring from home.” This approach enhances patient participation, reduces hospital visits, alleviates pressure on medical and nursing staff, conserves medical resources, and truly makes rehabilitation assessment intelligent and universal.

### 4.3 Limitations and future directions

Although the KROMNet model demonstrates impressive accuracy in assessing knee ROM, several limitations should be considered. A key limitation is its reliance on high-quality images for both training and testing. Factors such as image resolution, lighting conditions, and patient positioning can influence the model’s performance, especially in real-world, less controlled clinical settings. To address this, future work should focus on improving image preprocessing techniques, such as automated adjustments for varying lighting and patient positioning, thereby enhancing the model’s robustness and reliability across diverse settings. Another limitation is the lack of external validation across various hospitals and patient populations. Although the KROMNet model performed well within our cohort, further validation through multi-center studies involving a broader demographic range is essential. This would ensure the model’s generalizability and effectiveness across different clinical contexts. The current system also does not account for other variables that may influence knee recovery, such as age, comorbidities, and surgical techniques. These factors may significantly influence rehabilitation progress and could be incorporated into future iterations of the model. We plan to integrate these clinical variables to enhance the model’s accuracy and utility, offering a more comprehensive assessment of knee rehabilitation. In addition to improving the existing model, we are actively planning to expand our research into knee angle prediction. Currently, the model classifies ROM into categories according to predefined thresholds. In the future, we aim to develop a continuous knee angle prediction model that provides more precise assessments. This enhancement would address the model’s current limitation of categorical classification and offer more granular insights into a patient’s rehabilitation progress. By predicting specific knee angles, we aim to provide clinicians with a more detailed understanding of patients’ recovery trajectories, thereby improving postoperative care.

In conclusion, although the KROMNet model represents a promising tool for assessing knee ROM during postoperative rehabilitation, there are several areas for improvement. We are committed to advancing these areas through ongoing research that will address existing limitations and further enhance the clinical utility of our model.

## 5 Conclusion

In conclusion, this study demonstrates that KROMNet offer a highly accurate and efficient solution for monitoring knee ROM in post-TKA patients. The proposed method provides several advantages over traditional ROM assessment techniques and other advanced evaluation techniques, including improved accuracy, scalability, cost-effectiveness, and simplicity. Despite its limitations, the model holds great potential to transform postoperative care by simplifying the assessment process for clinicians and allowing patients to self-assess their recovery at home, ultimately improving patient outcomes and healthcare efficiency.

## Data Availability

The original contributions presented in the study are included in the article/Supplementary Material, further inquiries can be directed to the corresponding authors.

## Supplementary material

SUPPLEMENTARY FIGURE S1: Schematic diagram of the patient's flexion and extension. (a) 0°. (b) 25°. (c) 50°. (d) 75°. (e) 100°. (f) 125°.

SUPPLEMENTARY FIGURE S2: Preprocessing results of patient flexion and extension images. (a) 0°. (b) 25°. (c) 50°. (d) 75°. (e) 100°. (f) 125°.
