# Supplementary figures and images for: Using deep networks for knee range of motion monitoring in total knee arthroplasty rehabilitation
Source: Front Bioeng Biotechnol. 2025 Nov 19;13:1691591. doi: 10.3389/fbioe.2025.1691591 (PMC12673327; doi:10.3389/fbioe.2025.1691591)

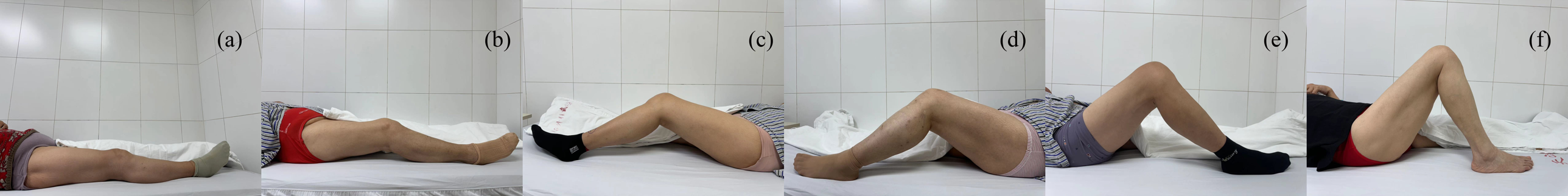

Supplement: Supplementary file 1 [file Image1.tiff]

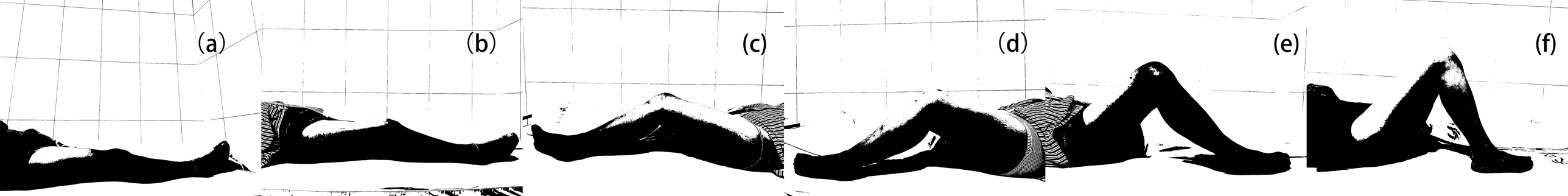

Supplement: Supplementary file 2 [file Image2.tiff]
